# Bio-Based Phthalonitrile Bearing a Schiff Base Moiety as a High-Performance Modifier for Epoxy Resins: Curing Behavior and Thermal Properties

**DOI:** 10.3390/ma19204324

**Published:** 2026-10-14

**Authors:** Cong Peng, Chenxu Liu, Zhanjun Wu

**Affiliations:** 1 College of Fiber Engineering and Equipment Technology, Jiangnan University, Wuxi 214122, China; pc@jiangnan.edu.cn (C.P.);; 2 School of Materials Science and Engineering, Dalian University of Technology, Dalian 116024, China

**Keywords:** epoxy, phthalonitrile, thermal stability, curing kinetics, viscosity

## Abstract

The thermal stability of epoxy resin (EP) constitutes one of the major bottlenecks restricting its high-temperature applications. Bio-based phthalonitrile resin (FUPN) bearing a Schiff base unit was utilized as a reactive modifier for a diglycidyl ether of bisphenol F (DGEBF)/diaminodiphenyl sulfone (DDS) system. The effects of FUPN on the thermal performance of cured epoxy networks were investigated. The glass transition temperature (*T*_g_) of the cured thermosets increased from 161 °C for neat DGEBF/DDS (EP/FU0) to 231 °C for EP/FU50. The char yield at 800 °C under N2 atmosphere rose from 21.4 wt% to 57.7 wt%, while the maximum mass loss rate decreased from 14.3%/min to 3.7%/min. The char yield under air atmosphere at 800 °C was remarkably enhanced from 3.4 wt% to 31.5 wt%. The char residues were further characterized via XPS and Raman spectroscopy. The peak heat release rate and total heat release of the thermoset during combustion were reduced by 76.7% and 54.2%, respectively. Furthermore, the uncured mixture maintained a low viscosity (<2 Pa·s) at moderate temperatures even with a high loading of FUPN, which is highly favorable for practical engineering applications.

## 1. Introduction

Epoxy resins are widely employed in coatings, adhesives, and matrices for fiber-reinforced composites owing to their outstanding mechanical properties, adhesion, chemical resistance, and processing versatility [1,2,3]. The ultimate performance of cured epoxy systems is largely governed by the type of curing agent. Epoxy networks crosslinked with rigid aromatic amines such as 4,4′-diaminodiphenyl sulfone (DDS) generally exhibit high glass transition temperatures, making them important representatives of high-performance thermosetting matrices [4,5]. Nevertheless, conventional epoxy/amine crosslinked networks still suffer from insufficient thermal stability at elevated temperatures, especially under oxygen-containing atmospheres [6,7]. Volatile pyrolysis products generated during thermal degradation not only degrade high-temperature structural stability but also raise safety hazards under combustion or thermal exposure [1,8]. A straightforward strategy to mitigate these drawbacks is to incorporate modifiers into existing epoxy/amine systems via blending. This approach avoids redesigning the backbone structure of epoxy resins and facilitates formulation tuning and implementation, so it is widely adopted in practical manufacturing [9,10,11]. Common modifiers include inorganic particles and second-phase polymeric additives [11,12,13]. Such components can improve the thermal stability, char-forming ability, and flame retardancy of epoxy resins, yet the effectiveness is not guaranteed; it depends on the structure and loading of the modifier, its dispersion and compatibility within the epoxy matrix, as well as interfacial interactions [14,15,16]. Poor dispersion leads to particle agglomeration, while inadequate compatibility may induce phase separation. Furthermore, high loading or strong inter-component interactions can increase resin viscosity, which is unfavorable for mixing, casting, and fiber impregnation [13,17]. Accordingly, enhancing the thermal stability and char-forming ability of cured epoxy/amine networks, suppressing heat release, and avoiding substantial compromise to processability remains a critical challenge for the modification of high-performance epoxy resins.

Phthalonitrile resins constitute a class of high-temperature thermosets. The nitrile groups can form thermally stable nitrogen-containing heteroaromatic structures such as triazine, phthalocyanine and isoindoline, thereby endowing the cured networks with outstanding thermal stability and char-forming ability [18,19]. Existing studies have demonstrated that modifying epoxy resins with phthalonitrile is an effective strategy. Relevant studies have reported that phthalonitrile structures can significantly enhance the thermal stability of epoxy resins. In our previous work [20], bisphenol A-based phthalonitrile (BAPH) was added to the E51/DDS system. The char yield of the cured resins at 800 °C under nitrogen was increased from 16% to 59.6%. Qiu et al. synthesized an epoxy monomer PNDEP containing phthalonitrile moieties [20]. The cured PNDEP/DDM system exhibited a *T*_g_ of 214 °C, which was 32 °C higher than that of the DGEBA/DDM system (182 °C). It also possessed a char yield of 56% at 700 °C under nitrogen atmosphere, 3.5 times that of the latter. Notably, the cured resin achieved excellent flame retardancy with a UL-94 V-0 rating; the peak heat release rate (pHRR) and total heat release (THR) were reduced by 88.8% and 84.2%, respectively. Hu et al. prepared binary co-cured systems of benzimidazole-containing phthalonitrile (PNBI) monomer and E51 epoxy resin [10]. The glass transition temperatures *T*_g_ of cured PNBI/E51 blends exceeded 207 °C as determined by DMA. The temperature at 5% weight loss *T*_5%_ and char yield at 800 °C were above 360 °C and 14%, respectively, under nitrogen atmosphere.

In recent years, bio-based polymers derived from renewable biomass, including lignocellulose, vegetable oils, starch, and microbial fermentation products, have emerged as highly promising alternatives to fossil resources. Weng et al. [21] prepared phthalonitrile resins using natural magnolol and honokiol. The resulting thermosets exhibited ultra-high glass transition temperatures exceeding 500 °C, with a maximum *T*_5%_ of 527 °C, showing superior thermal performance compared with petroleum-based counterparts without allyl groups. Ma et al. [22] synthesized self-catalytic phthalonitrile monomers (FANP and IFANP) using biomass-derived vanillin, isovanillin, and furan-based furfurylamine. The obtained monomers possessed a low melting point of 120 °C and favorable viscosity, facilitating practical processing. Notably, the cured FANP-380 sample exhibited an initial storage modulus above 4000 MPa and a *T*_g_ higher than 400 °C.

Structural differences commonly exist between fossil-based and bio-based raw materials. For instance, bio-based units contain abundant highly polar groups such as methoxy and phenolic hydroxyl groups, whereas fossil-based epoxy resins exhibit relatively low molecular polarity. The furan rings and multi-substituted aromatic rings (eugenol-type) in many bio-based precursors possess rigidity distinct from that of the bisphenol A benzene rings. The mismatch in segmental rigidity gives rise to an unfavorable entropic contribution to miscibility. Therefore, the ultimate performance of fossil-based epoxy resins modified by bio-based phthalonitrile remains to be explored. On the basis of previous studies, this work used a reported bio-based phthalonitrile FUPN synthesized via vanillin and furfurylamine [22] as a modifier for the widely used fossil-based DGEBF/DDS system. The FUPN–epoxy co-curing interactions, and the correlation of these interactions with the thermal stability, char-formation ability and the processability will be investigated.

## 2. Experiment

### 2.1. Materials

Bisphenol F epoxy resin (DGEBF, epoxy value: 0.6 mol/100 g) was purchased from Nantong Xingchen Synthetic Material Co., Ltd. (Nantong, China). Vanillin (≥99% purity), 4-nitrophthalonitrile (4-Nph, ≥98% purity), N, N-dimethylformamide (DMF, ≥99% purity), furfurylamine (≥99% purity), 4,4′-diaminodiphenyl sulfone (DDS, ≥98% purity) and anhydrous sodium acetate (≥99% purity) were all obtained from Aladdin Biochemical Technology Co., Ltd. (Shanghai, China). Anhydrous ethanol was supplied by Sinopharm Chemical Reagent Co., Ltd. (Shanghai, China). All reagents were used as received unless otherwise specified.

### 2.2. Synthesis of Phthalonitrile Monomer Fupn

**Figure 1:**
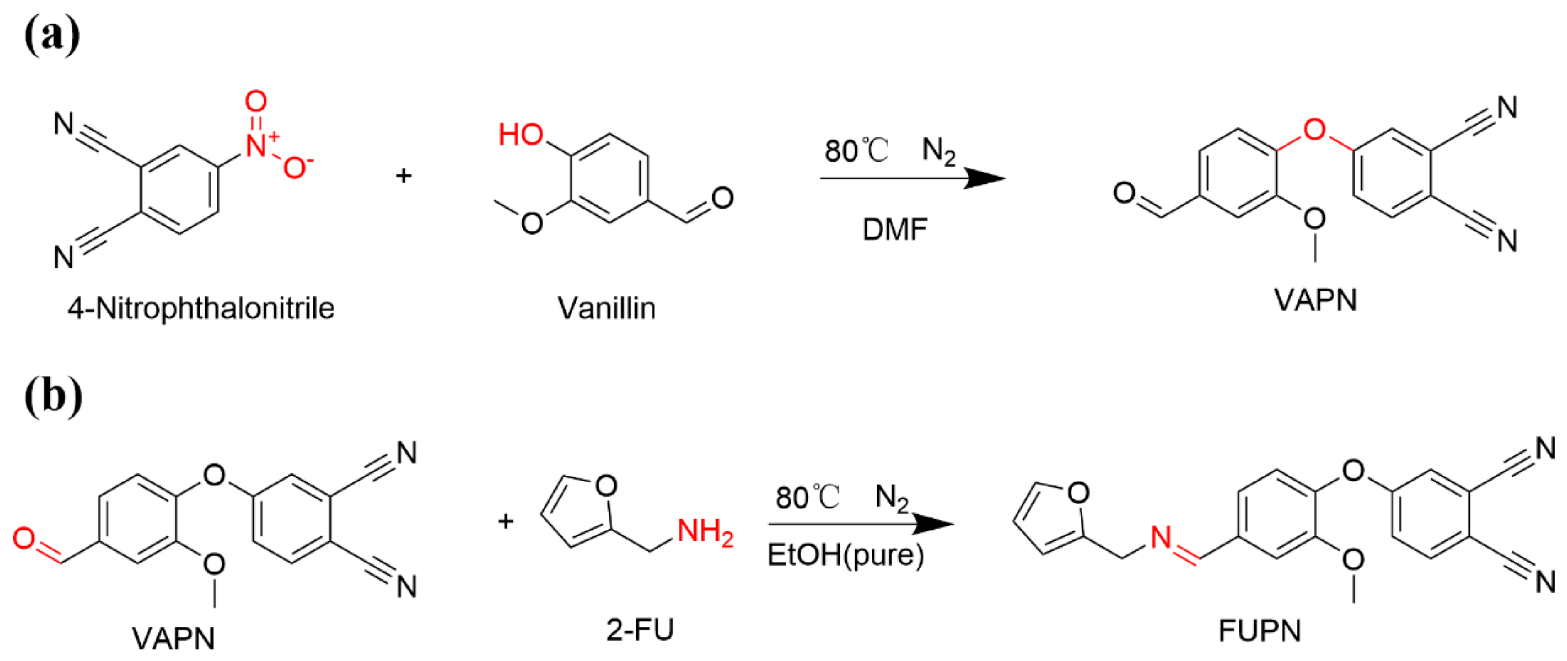
Synthetic routes to (**a**) intermediate VAPN and (**b**) final product FUPN.

The synthetic route of the bio-based phthalonitrile FUPN was carried out according to a previously reported procedure [22]. The detailed synthetic procedures are shown in Figure 1, and the corresponding results are shown in the Supplementary Materials.

### 2.3. Curing of the DGEBF/DDS/FUPN Blend

FUPN was incorporated into the DGEBF/DDS system to prepare EP/FUx cured resins. The detailed procedures are described as follows. FUPN and DGEBF were stirred continuously at 130 °C for 5 min. After adding DDS according to the epoxy value of DGEBF, the mixture was further stirred at the same temperature for 20 min until a pale yellow transparent liquid was obtained. Subsequently, the mixture was poured into a preheated stainless steel mold coated with a release agent, and degassed under vacuum in a vacuum oven at 130 °C for 30 min to remove air bubbles. A stepwise curing schedule was adopted: 180 °C/1 h, 200 °C/2 h, 220 °C/2 h, 255 °C/2 h, 265 °C/3 h and 280 °C/3 h.

The cured samples were designated as EP/FUx (x = 0, 20, 30, 40, 50), where x represents the mass fraction of FUPN in the total mass of DGEBF and FUPN. Stoichiometric DDS calculated based on the epoxy value of DGEBF was used for all EP/FUx formulations. EP/FU0 was cured using a curing schedule: 160 °C/2 h, 180 °C/4 h and 200 °C/2 h [23]. The formulation compositions of all samples are summarized in Table 1.

### 2.4. Characterization

The chemical structures of the synthesized monomers and cured resins were characterized by Fourier-transform infrared spectroscopy (FTIR, Thermo Fisher Nicolet iS10, Madison, WI, USA) over a wavenumber range of 4000–500 cm^−1^ with a resolution of 4 cm^−1^. The molecular structures of FUPN and its intermediate VAPN were further verified by ^1^H NMR and ^13^C NMR using DMSO-d_6_ as the solvent, with tetramethylsilane (TMS) as the internal standard.

Curing behavior was investigated by differential scanning calorimetry (DSC, Mettler DSC 3 +, Greifensee, Switzerland) under a nitrogen atmosphere. Sample mass was approximately 5–10 mg, and the scanning temperature range was 50–400 °C. FUPN and uncured EP/FU40 were tested at heating rates of 5, 10, 15 and 20 K/min; all other specimens were measured at 10 K/min.

Temperature–viscosity curves of EP/Fux were acquired using a rotational rheometer (Thermo Fisher Scientific, Waltham, MA, USA). The test was performed at a fixed frequency of 1 Hz and a heating rate of 5 K/min, and terminated once gelation set in.

Thermogravimetric analysis (TGA, Mettler TGA2, Nänikon, Switzerland) was carried out under nitrogen and air atmospheres. Approximately 10 mg of sample was heated from room temperature to 800 °C at 10 °C/min. The T_5_%, Tmax, Rmax and char residue at 800 °C were derived from the TGA/DTG curves.

Tube-furnace thermal treatment was conducted on cured specimens with comparable dimensions (ca. 5 mm × 5 mm × 2 mm). The samples were heated to 800 °C at 10 °C/min under nitrogen, and the morphology of char residues was observed after cooling.

For microscale combustion calorimetry (MCC) measurements, the flow rates of oxygen and nitrogen were set to 20 and 80 cm^3^/min, respectively. The peak heat release rate (pHRR), total heat release (THR) and corresponding peak temperature were obtained from heat release rate curves.

X-ray photoelectron spectroscopy (XPS) measurements were performed using a Thermo Scientific Escalab 250 Xi spectrometer (Thermo Fisher Scientific, Waltham, MA, USA) equipped with a monochromatic Al Kα X-ray source (1486.6 eV). The binding energies of both survey and high-resolution spectra were calibrated using the C 1s peak at 284.8 eV as an internal reference.

Raman spectra were acquired using an inVia Reflex micro-Raman spectrometer with a 633 nm excitation laser. The laser power at the sample surface was 5 mW, and a 50× objective was used. The acquisition time was 10 s per scan with three accumulations. The Raman shift was calibrated against the 520 cm^−1^ peak of single-crystalline silicon.

After grinding and polishing the cross-sections of cured samples, height and phase images were captured using atomic force microscopy (AFM, Thermo Fisher Scientific, Waltham, MA, USA) in tapping mode.

Dynamic thermomechanical properties were measured with a DMA Q800 instrument (TA Instruments, New Castle, DE, USA) in single-cantilever mode. Specimen dimensions were 30 mm × 10 mm × 3 mm, the test frequency was 1 Hz, and the heating rate was 5 °C/min. *T*_g_ was determined from the tan δ peak temperature, and the apparent crosslink density was estimated from the storage modulus E’ at (*T*_g_ + 30).

## 3. Results and Discussion

### 3.1. Structural Confirmation of the Products

The chemical structures of VAPN and FUPN were characterized by FTIR, as shown in Figure S1. 4-nitrophthalonitrile (4-Nph) exhibits a characteristic absorption peak of –C≡N at approximately 2233 cm^−1^, as well as asymmetric and symmetric stretching vibration peaks of –NO_2_ near 1536 and 1353 cm^−1^, respectively. Compared with 4-Nph, the characteristic –NO_2_ peaks of VAPN significantly weakened or disappeared, whereas the –C≡N band remains, indicating successful substitution of the nitro group without destruction of the phthalonitrile framework. The aldehyde C=O peak around 1670–1700 cm^−1^ was still observable in VAPN, demonstrating that the aldehyde group was preserved during the first-step reaction. After further condensation with furfurylamine, the aldehyde C=O peak in FUPN decreases substantially or vanished, while a new absorption band assigned to the Schiff-base C=N linkage appeared near 1646 cm^−1^. The characteristic N–H stretching vibration of primary amine –NH_2_ in furfurylamine was no longer evident in FUPN, revealing the consumption of –NH_2_. The –C≡N peak persisted in FUPN, confirming that the phthalonitrile curing sites were retained.

**Figure 2:**
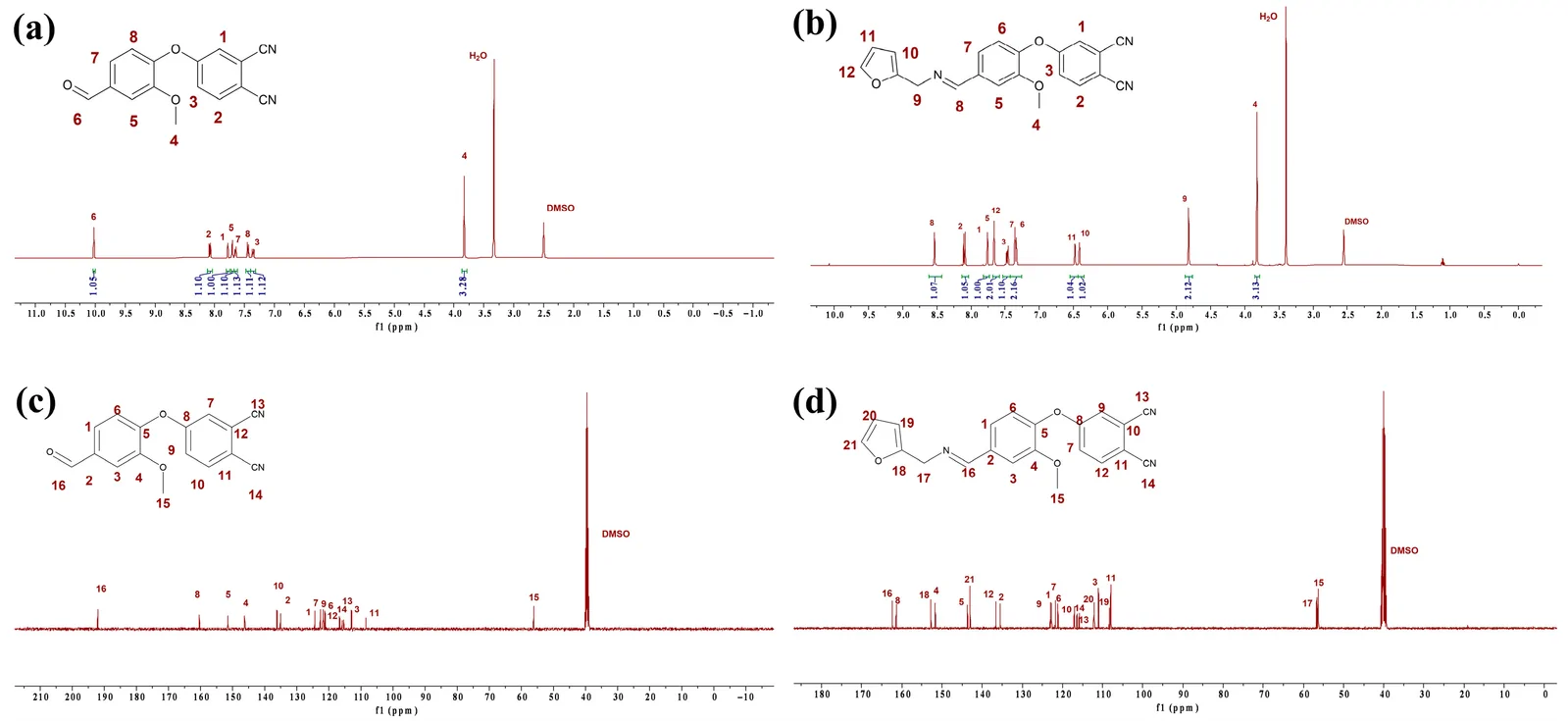
(**a**,**b**) ^1^HNMR and (**c**,**d**) ^13^CNMR spectra of VAPN and FUPN.

The chemical structures of VAPN and FUPN were further confirmed by ^1^H NMR and ^13^C NMR, as shown in Figure 2. VAPN shows an aldehydic proton at δ 10.02 ppm and a methoxy singlet at δ 3.83 ppm. After condensation with furfurylamine, the aldehydic proton signal disappeared in FUPN, while a new Schiff-base –CH = N– proton signal emerged at δ 8.48 ppm. The signals at δ 6.43 and 6.36 ppm can be assigned to furan ring protons, and the singlet at δ 4.77 ppm corresponds to –CH_2_–. In the ^13^C NMR spectra, the aldehyde carbon signal of VAPN at δ 192.02 ppm vanished in FUPN, accompanied by the appearance of carbon signals related to the imine structure and furfurylamine moiety. The nitrile carbon signals near δ 115–117 ppm were retained in both VAPN and FUPN. Combined with the FTIR and NMR results, the chemical structure of the target product FUPN was verified.

### 3.2. Curing Analysis

Non-isothermal DSC and the Starink isoconversional method were employed to investigate the curing kinetics of FUPN; detailed calculation procedures are provided in Section S2 of the Supplementary Materials. The non-isothermal DSC curves of VAPN and FUPN are displayed in Figure S2. A melting endotherm near 180 °C was observed for intermediate VAPN, while no curing exotherm appeared within the tested temperature range. In contrast, FUPN exhibited a melting peak around 120 °C accompanied by a distinct curing exotherm at 280 °C. The depression of melting temperature arises mainly from two factors. First, the strongly polar terminal aldehyde group of VAPN is replaced by an imine group, which weakens intermolecular dipole–dipole interactions. Second, the introduced furan ring adopts a nonplanar conformation and imposes considerable steric hindrance, hindering molecular packing. Most phthalonitrile monomers undergo crosslinking above 350 °C [24,25,26], which explains the absence of a crosslinking exotherm in the DSC trace of VAPN. The Schiff-base group incorporated into FUPN contains a lone electron pair on the nitrogen atom; this nitrogen acts as a nucleophile to activate the crosslinking reaction of cyano groups, thereby substantially lowering the crosslinking temperature of FUPN [27,28]. The reduced melting point and curing temperature are favorable for practical engineering applications of FUPN.

**Figure 3:**
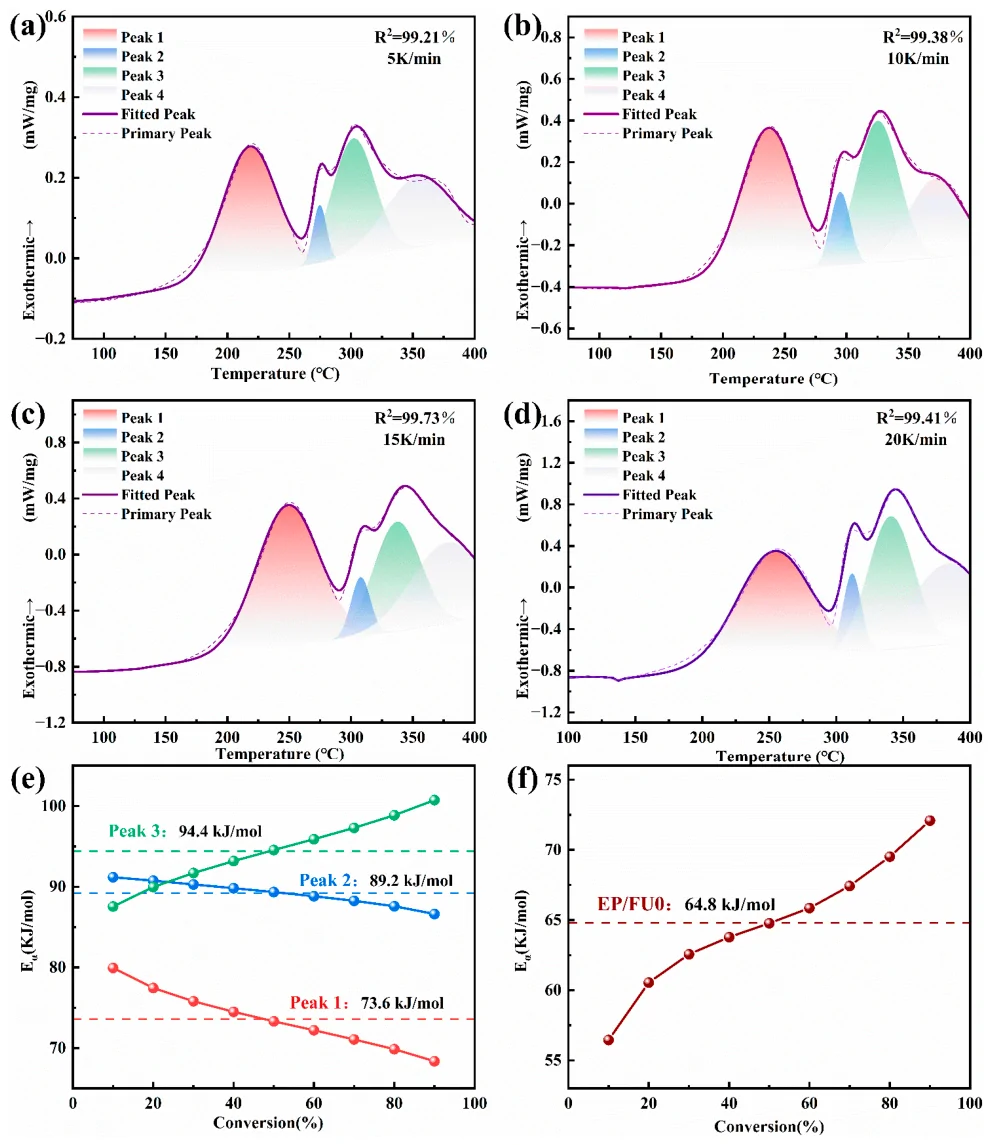
Deconvolution results of DSC curves for curing process of EP/FU40 at heating rates of (**a**) 5, (**b**) 10, (**c**) 15 and (**d**) 20 K/min and (**e**) the activation energy *E_α_* corresponding to peak1, peak2, peak3 as functions of conversion; (**f**) the activation energy *E_α_* of the crosslink reaction of EP/FU0 as a function of conversion.

A series of DGEBF/DDS blends with different FUPN loadings were fabricated in this work. The DSC curves of EP/FU40 blend measured at various heating rates were deconvoluted, and all *R*^2^ values exceeded 0.99, as presented in Figure 3. No obvious melting endotherm was observed near 120 °C for the blended system, which can be attributed to favorable compatibility between DGEBF and FUPN; DGEBF molecules restrain the crystallization tendency of FUPN. Four independent exothermic peaks were resolved from the DSC curve of EP/FU40 from low to high temperature. Peak 1 is assigned to the crosslinking reaction between DGEBF and DDS [23]. Combined with subsequent TGA results, peak 4 can be ascribed to the exothermic event arising from thermal degradation of weakly crosslinked epoxy fractions at high temperatures [23,29]. Notably, the deconvolution results revealed two exothermic peaks associated with the crosslinking of FUPN (peak 2 and peak 3), which differs from the single exothermic band of neat FUPN in Figure S2. Similar two-stage curing behavior has been reported for phthalonitrile/epoxy blends [30,31,32].

**Figure 4:**
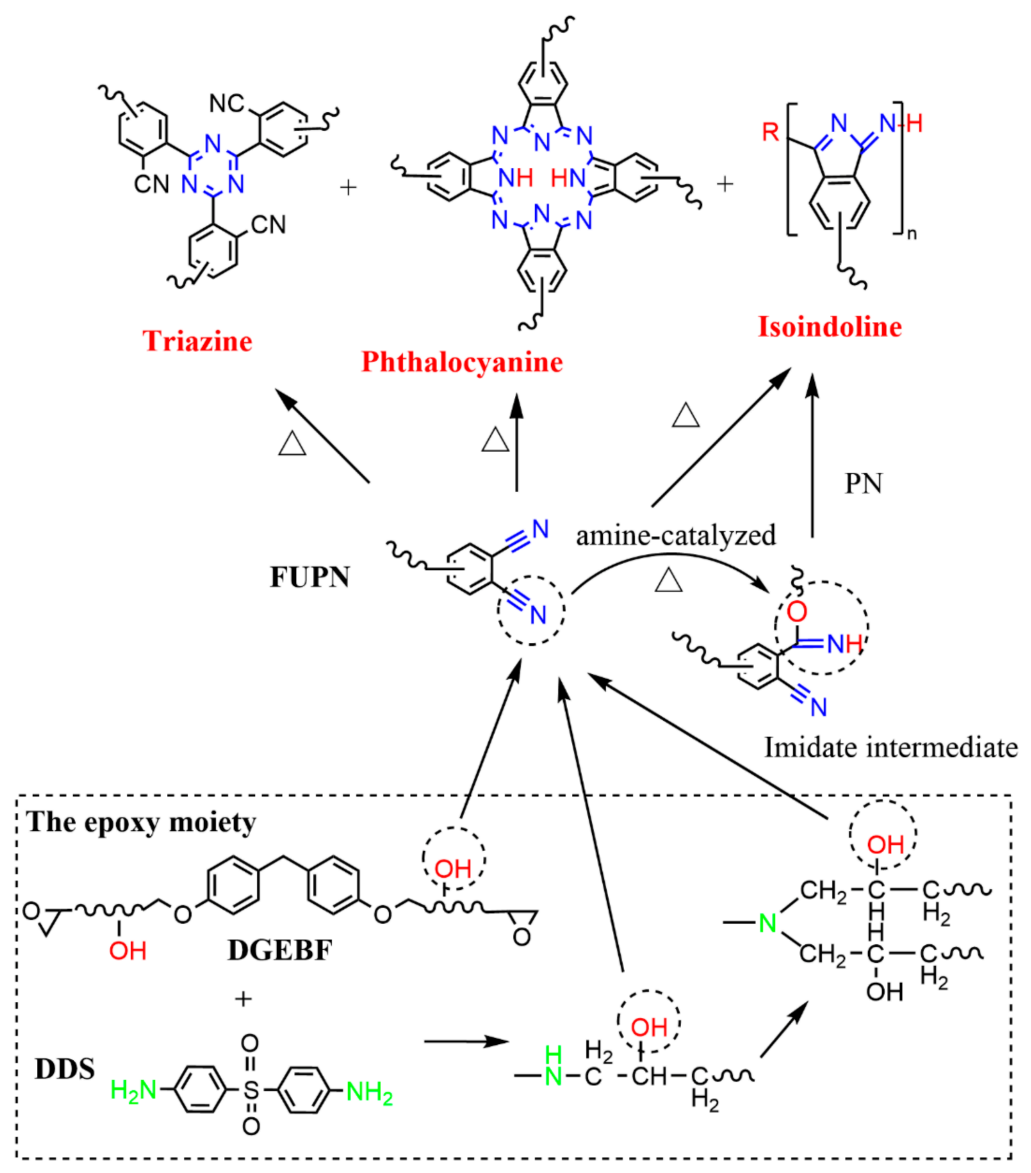
Proposed reaction pathway of cyano groups with crosslinked DGEBF/DDS products.

The DSC curing curves of different combinations of DGEBF, DDS and FUPN are shown in Figure S3. The curing of FUPN in both FUPN/DGEBF and EP/FU40 systems exhibits a bimodal exothermic profile. For FUPN/DGEBF blend, the left peak (290 °C) appears at the temperature nearly identical to peak 2 of EP/FU40 and the single peaks of neat FUPN and FUPN/DDS mixtures (Figure S3a), confirming that peak 2 corresponds to conventional phthalonitrile crosslinking. The right peak (342 °C) emerges at the temperature 18 °C higher than that of peak 3 (324 °C). In addition, for FUPN/DGEBF, the intensity of the left peak is markedly higher than that of the right peak, which is opposite to the trend between peak 2 and peak 3 of EP/FU40. The proposed reaction mechanism for peak 3 is illustrated in Figure 4. Cyano groups are likely to react with secondary hydroxyl groups in DGEBF moiety to form imidate intermediates, which subsequently undergo cyclization with ortho cyano groups and intermolecular polymerization to generate poly-isoindoline structures. When both DGEBF and DDS are present, the amine species may serve as a catalyst, lowering the temperature of peak 3 (compared with that of the right peak of the FUPN/DGEBF blend). Meanwhile, the crosslinked network formed by DGEBF/DDS suppresses the crosslinking pathway corresponding to peak 2, leading to the observation that peak 3 is stronger than peak 2.

Moreover, the activation energy *E*_α_ of the crosslink reaction related to peak1–peak3 as well as that of EP/FU0 (i.e., DGEBF/DDS) were calculated via the Starink isoconversional method, as shown in Figure 3e,f. The average *E*_α_ corresponding to the crosslink between DGEBF and DDS in EP/FU40 (peak 1) was 73.6 kJ/mol, which was close to that of EP/FU40 (64.8 kJ/mol). This indicates that the incorporation of FUPN has a negligible effect on the crosslinking of DGEBF and DDS. The average *E*_α_ related to the crosslink of phthalonitrile was 89.2 kJ/mol and 94.4 kJ/mol, respectively, corresponding to peak 2 and peak 3, which were relatively higher.

The DSC data of EP/FUx with different FUPN loadings are presented in Figure S4 and Table S1. The area ratio of peak 2 to peak 3 was adopted to evaluate the relative contributions of the self-curing of FUPN and the FUPN–DGEBF interaction. With the increase of FUPN loading, the area ratio decreases rapidly at first and then declines gradually. It can be inferred that the change in the ratio of peak 2 to peak 3 can be attributed to the variation in the aggregation state of FUPN molecules. FUPN molecules can form ordered aggregates via π–π stacking of aromatic rings and dipole–dipole interactions among polar Schiff-base and nitrile groups. At low FUPN loading (EP/FU20), FUPN molecules possess sufficient free volume and mobility in the medium and readily form ordered pre-associated aggregates, which favors the self-cyclotrimerization (peak 2) of FUPN. Upon increasing FUPN loading, the reduced free volume restricts the formation of ordered pre-assemblies. Consequently, the relative proportion of peak 3 increases.

**Figure 5:**
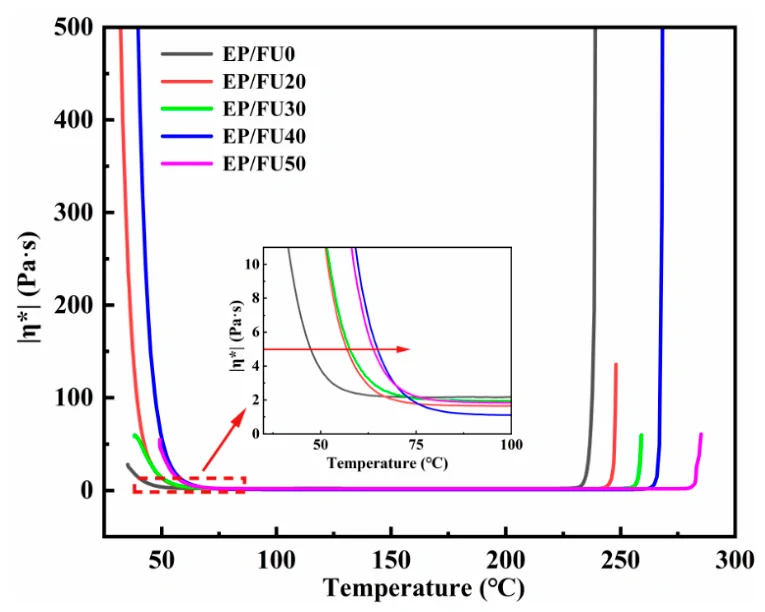
Temperature–viscosity curves of EP/FUx blends.

Resin viscosity and low-viscosity temperature window are important criteria for evaluating the processability of thermosetting resins [33]. Dynamic temperature-ramp rheological measurements were performed to characterize the rheological behavior of the blended systems, as shown in Figure 5. Even at high FUPN loadings, favorable flowability can be achieved at moderate and low temperatures (EP/FU50 reached a viscosity of 5 Pa·s at 64 °C). All formulations exhibit a relatively wide low-viscosity temperature window (around 1–2 Pa·s), indicating that the incorporation of FUPN exerts no obvious adverse effects on processing, and these blends meet the technical requirements for thermal blending, degassing and casting.

### 3.3. Properties of the Cured Resins

**Figure 6:**
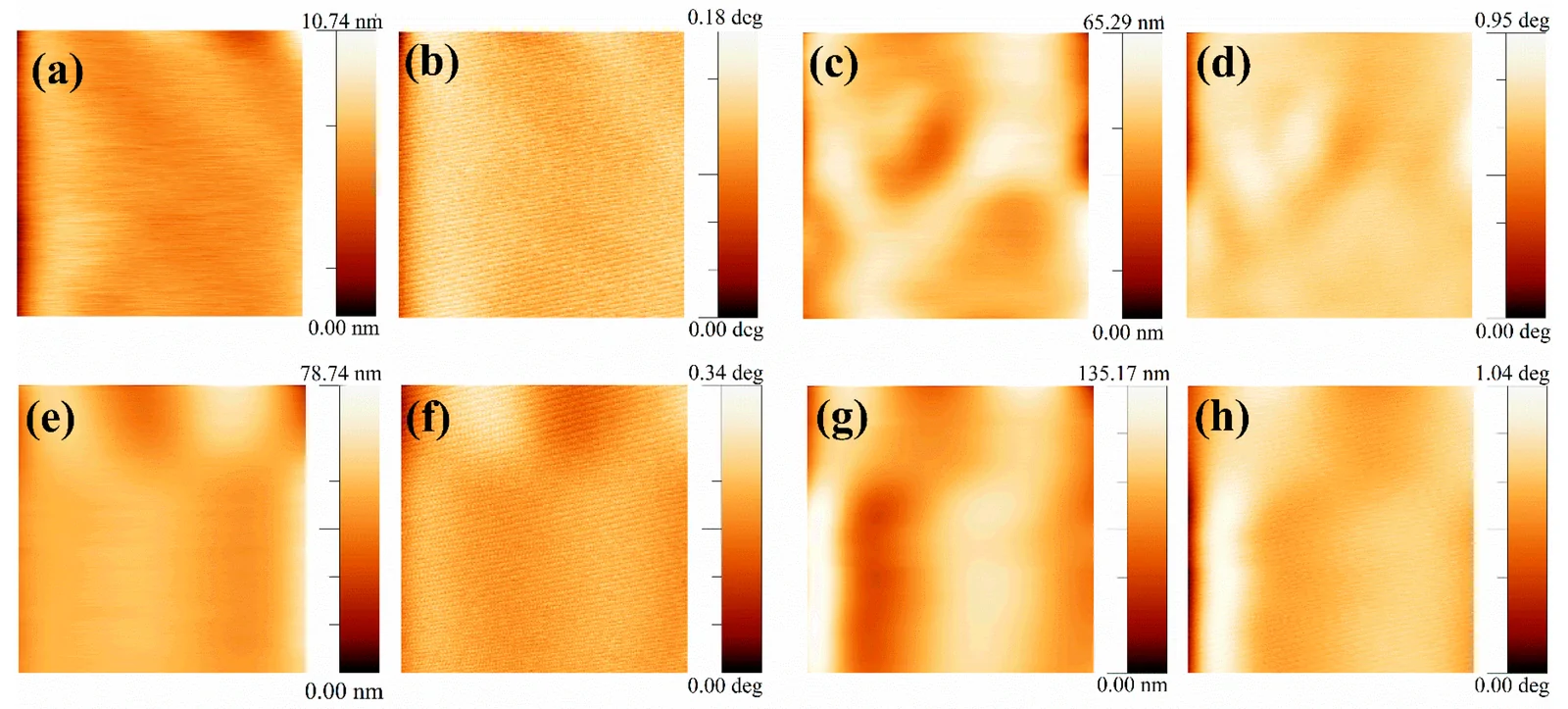
AFM height and phase images of cured EP/FUx resins; all scans were performed over a 5 μm × 5 μm area. (**a**,**b**) EP/FU20, (**c**,**d**) EP/FU30, (**e**,**f**) EP/FU40, (**g**,**h**) EP/FU50.

Atomic force microscopy (AFM) was employed to investigate whether the incorporation of FUPN induces obvious phase separation during curing. As shown in Figure 6, the phase images of EP/FUx exhibit continuous contrast variation without distinct sea-island structures or sharp phase boundaries. This indicates that bio-based FUPN possesses favorable compatibility with conventional petroleum-based DGEBF, and no large-scale phase separation occurs. This observation is consistent with the DSC result that no melting endotherm of FUPN is detected in the blends, collectively demonstrating that FUPN is miscible with the DGEBF/DDS system at the molecular scale.

**Figure 7:**
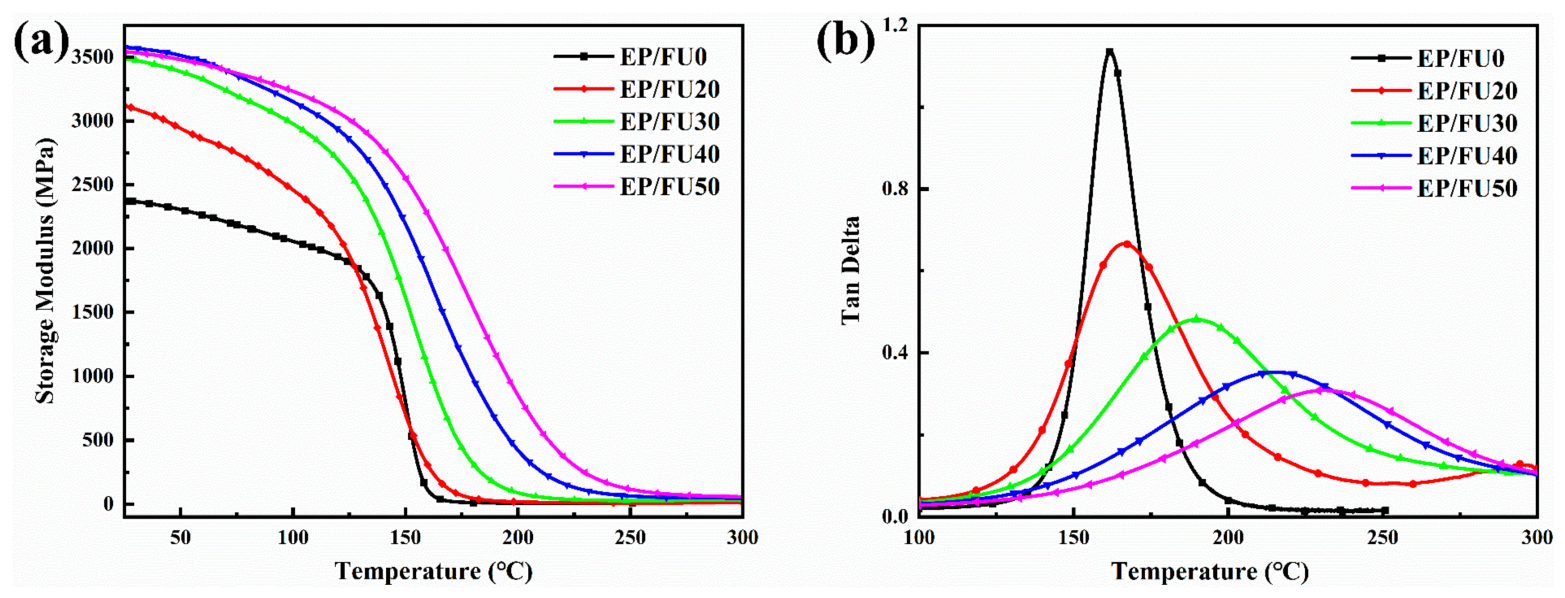
Temperature dependence of (**a**) storage modulus and (**b**) loss tangent obtained from dynamic mechanical analysis (DMA).

DMA measurements were carried out to investigate how the incorporation of phthalonitrile moieties affects the storage modulus (*E*′) and glass transition temperature (*T*_g_) of cured materials. The temperature at the peak of loss tangent is commonly taken as the *T*_g_ of a material, as shown in Figure 7 and Table 2. The introduction of FUPN increases the *T*_g_ of cured resins in a regular trend. The *T*_g_ rises from 161 °C (EP/FU0) to 231 °C (EP/FU50). Storage modulus (*E*′) reflects the stiffness of materials; a higher value indicates a harder material with greater resistance to deformation. With increasing FUPN loading, the storage modulus at room temperature (*E*′_25_, 25 °C) and at moderate high temperature (*E*′_100_, 100 °C) both increase remarkably. For EP/FU50, E′_25_ and E′_100_ reach 3542 MPa and 3232 MPa, respectively. The modulus retention ratio (*R_E_*′ = *E*_100_*/E*_25_) is 91.25% upon heating to 100 °C. Furthermore, the rubbery-state storage modulus (*E*′_r_, measured at *T*_g_ + 30 K) increases monotonically. The *E*′_r_ of EP/FU50 (88 MPa) is substantially higher than that of EP/FU0 (10 MPa). The variations in *T*_g_ and *E*′ can be attributed to the aromatic rigid backbone of FUPN. After curing, highly crosslinked heterocyclic structures (e.g., phthalocyanine, triazine, isoindoline) are formed. Uniform blending of all components enables the crosslinked FUPN framework to act as an effective reinforcing phase, restricting the segmental motion of the DGEBF/DDS network and yielding a higher crosslink density of the cured resins [34,35]. The apparent crosslink density can be calculated by Equation (1):

(1) $$\nu_{e}=\frac{{E\prime }_{r}}{3RT}$$

where *R* (gas constant) = 8.314 J·mol^−1^·K^−1^, and *T* = (*T*_g_ + 30) K.

**Figure 8:**
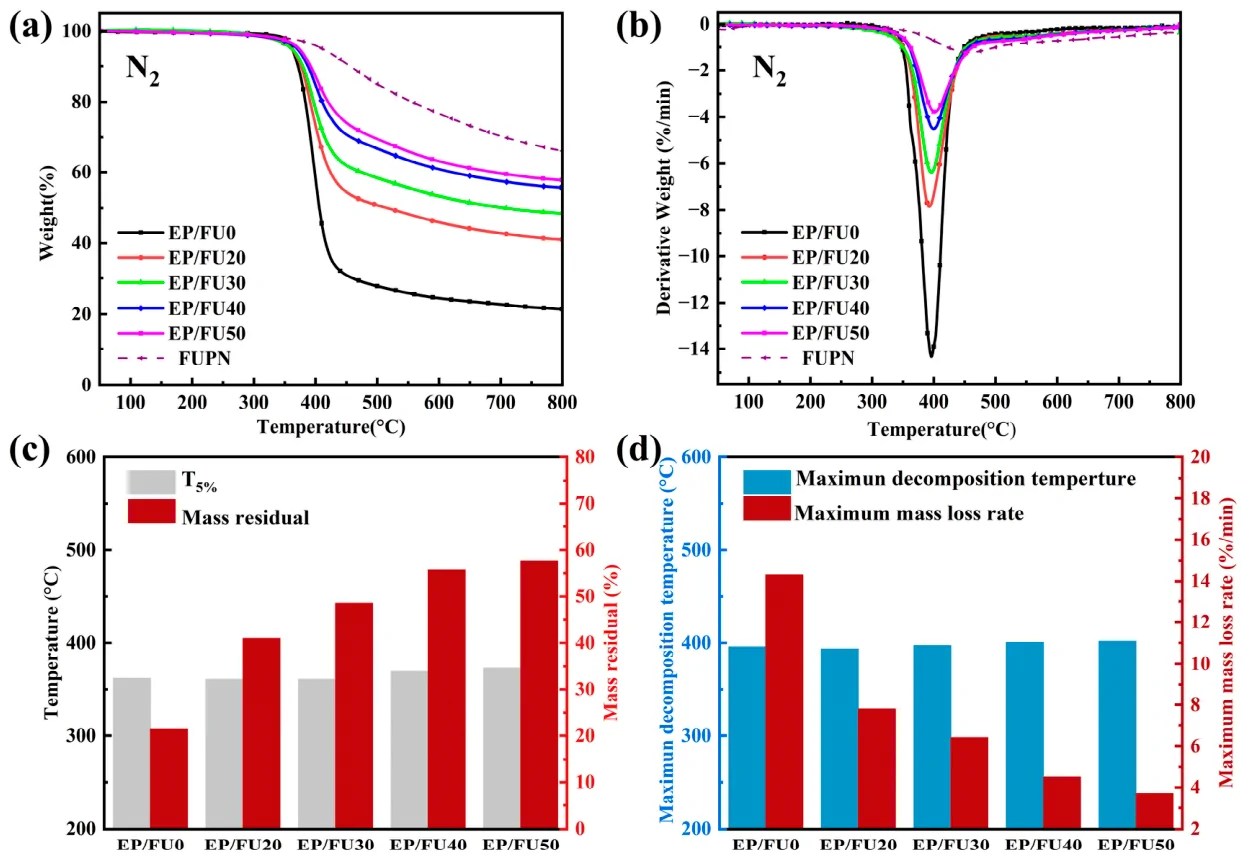
(**a**) TGA curves, (**b**) DTG curves, (**c**) T_5%_ and residual mass at 800 °C, (**d**) the maximum mass losing rate and the corresponding temperature of cured EP/FUx resins under N_2_ atmosphere.

TGA was utilized to investigate the thermal stability of EP/FUx blends. Figure 8 and Table 3 display the TGA/DTG curves and characteristic parameters of all cured samples under a nitrogen atmosphere. The *T*_5%_ of cured EP/FUx resins ranges from 361 to 373 °C; with increasing FUPN loading, *T*_5%_ rises slightly compared with the value of 362 °C for EP/FU0. TGA data of neat FUPN indicate that negligible mass loss of the crosslinked FUPN network occurs within this temperature range, and the mass loss of the blended cured resins mainly originates from degradation of the DGEBF/DDS network. It can be inferred that the incorporation of FUPN does not exert an obvious adverse effect on the epoxy crosslinked network.

A more pronounced effect of FUPN is the enhancement of final char yield *C*_exp_ and the reduction of maximum mass loss rate *R*_max_. The improvement is most significant for EP/FU50: *C*_exp_ increases from 21.4 for EP/FU0 to 57.7, while *R*_max_ decreases from 14.3%/min (EP/FU0) to 3.7%/min. Assuming each component decomposes independently without interaction, the theoretical char yield of the blend can be estimated by the proportional rule shown in Equation (2).

(2) $$C_{\mathrm{cal}}=w_{\mathrm{EP}}C_{\mathrm{EP}}+w_{\mathrm{FU}}C_{\mathrm{FU}}$$

where *C_cal_* denotes the theoretical char yield of the blend, and *C*_EP_, *C*_FU_, *w*_EP_ and *w*_FU_ represent the char yields and mass fractions of the epoxy component and phthalonitrile component, respectively.

As listed in Table 3, the experimentally measured char yield values are markedly higher than the calculated results, implying potential interactions between the two components. The above results demonstrate that the high char-forming capacity of FUPN further protects the epoxy crosslinked network and mitigates mass loss during thermal decomposition.

**Figure 9:**
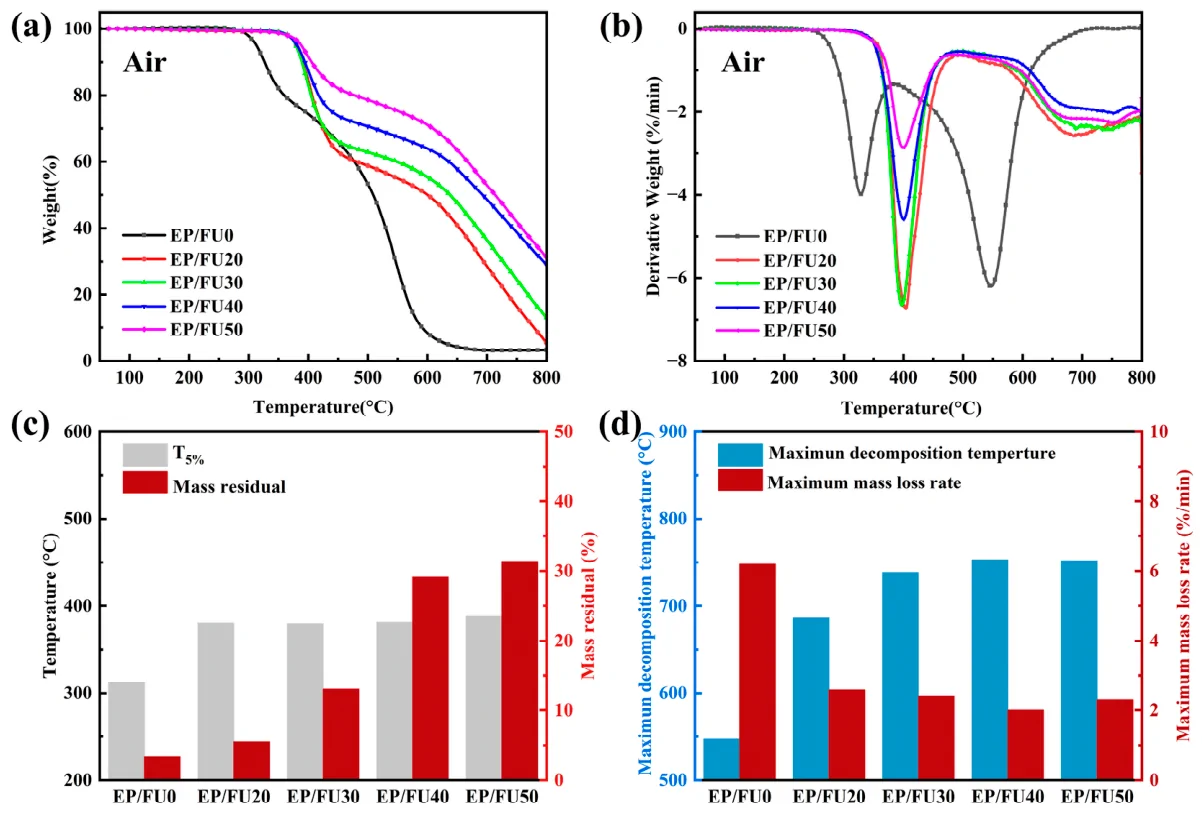
(**a**) TGA curves, (**b**) DTG curves, (**c**) T_5%_ and char yield at 800 °C, (**d**) the maximum mass losing rate and the corresponding temperature of cured EP/FUx resins under air atmosphere.

The thermo-oxidative decomposition behavior of cured resins was further investigated under an air atmosphere. As shown in Figure 9a,b and Table 4, all systems exhibit two distinct mass-loss stages. The first stage mainly corresponds to the pyrolysis of the resin network, whereas the second stage is associated with further oxidation of pyrolysis products and char layers [36]. Severe oxidative decomposition occurs for EP/FU0 in air. After incorporating FUPN, *T*_5%_ increases from 312.6 °C up to 387.8 °C, and *T*_1max_ rises from 328.5 °C to 404.1 °C, demonstrating a remarkable delay in initial thermal decomposition. In the oxidative degradation stage, *T*_2max_ increases from 546.9 °C to 752.9 °C, while *R*_2max_ decreases from 6.2%/min to 2.0%/min, revealing that the high-temperature oxidative degradation is significantly suppressed. Cured epoxy resins typically exhibit near-zero char yield at 800 °C under air. Notably, EP/FU50 exhibited a residual mass of 31.5% at 800 °C under air, a value considerably higher than those of most reported multifunctional or modified thermally stable epoxy resins, as shown in Table 5. This phenomenon is likely attributed to oxidative coupling of the Schiff-base moieties in FUPN upon high-temperature oxidation (e.g., crosslinking with unreacted cyano groups), forming thermally stable fused-ring structures containing C=N, C–N or oxacyclic units. Such structures possess superior resistance to high-temperature oxidation and therefore elevate the final char yield of the material [37].

FUPN adopted in this work is a monofunctional monomer, which theoretically affords a lower crosslink density compared with bifunctional phthalonitriles. Nevertheless, the FUPN-modified epoxy system achieved superior thermal stability to those modified with bifunctional phthalonitriles reported in the literature. In fact, for bifunctional phthalonitriles in the formed epoxy/amine crosslinked network, it is difficult for cyano groups at both terminals to participate in crosslinking simultaneously. Once crosslinking occurs at one terminal, the molecular mobility is largely locked, and the cyano group at the other terminal can hardly undergo further crosslinking. Although FUPN bears only one phthalonitrile moiety, its smaller molecular volume may facilitate the formation of additional phthalonitrile crosslinks within the epoxy/amine network and construct a more favorable molecularly interpenetrated network. Accordingly, the thermal stability of the composite material was greatly enhanced.

**Figure 10:**
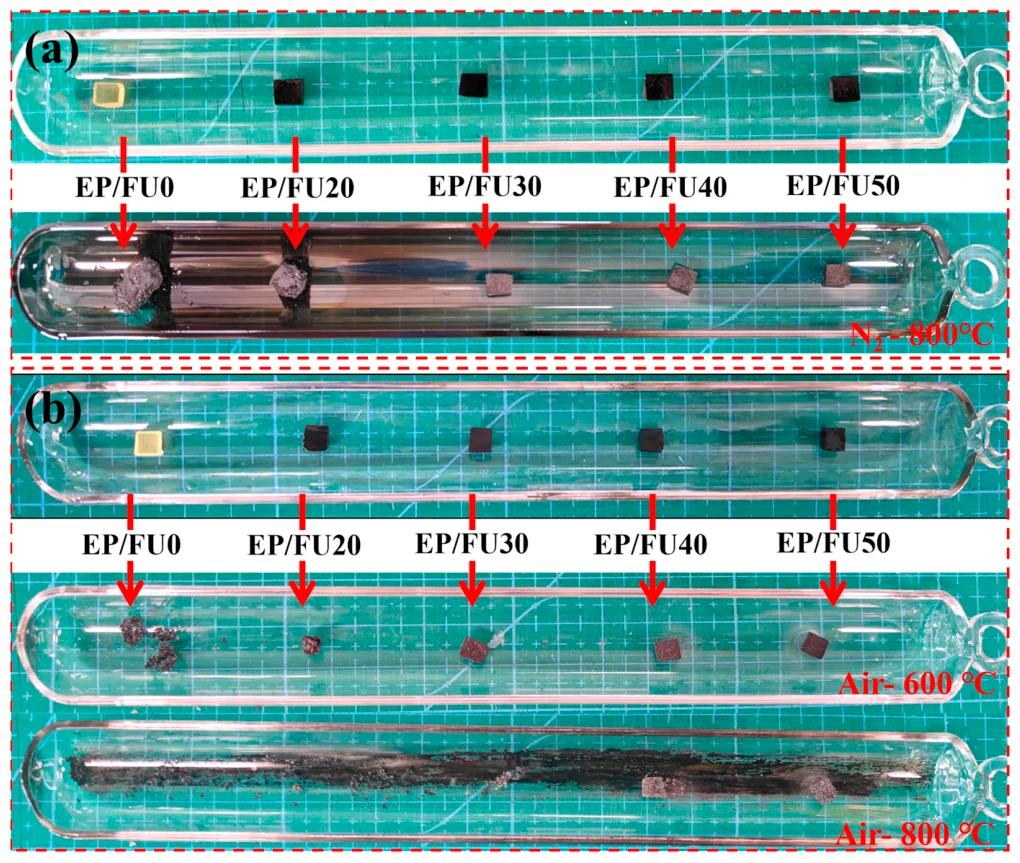
Digital photographs of char residues for various samples after pyrolysis in a tube furnace: (**a**) in N_2_ atmosphere, (**b**) in air atmosphere.

The cured samples were heated to 800 °C at a heating rate of 10 °C/min under N_2_/air atmospheres in a tube furnace to obtain char residues. The specimens were promptly removed from the tube and photographed upon reaching the set temperature. The appearance of the char yields are shown in Figure 10. For EP/FU0 under nitrogen, the appearance of the residue differed significantly from that of the original sample; obvious melting and swelling features were observed after heating, indicating the collapse of the crosslinked molecular framework during heating. After thermal treatment at 800 °C in air, its shape was completely destroyed, leaving only a small amount of debris. Under both N_2_ and air atmospheres, the resistance to thermal decomposition of the cured resins increased with rising FUPN content. Notably, EP/FU50 still retained its original contour after treatment at 800 °C in air, which is rarely observed in epoxy-based thermosets. These observations are consistent with the TGA results and demonstrate that FUPN-modified epoxy resins possess outstanding thermal and thermo-oxidative stability.

**Figure 11:**
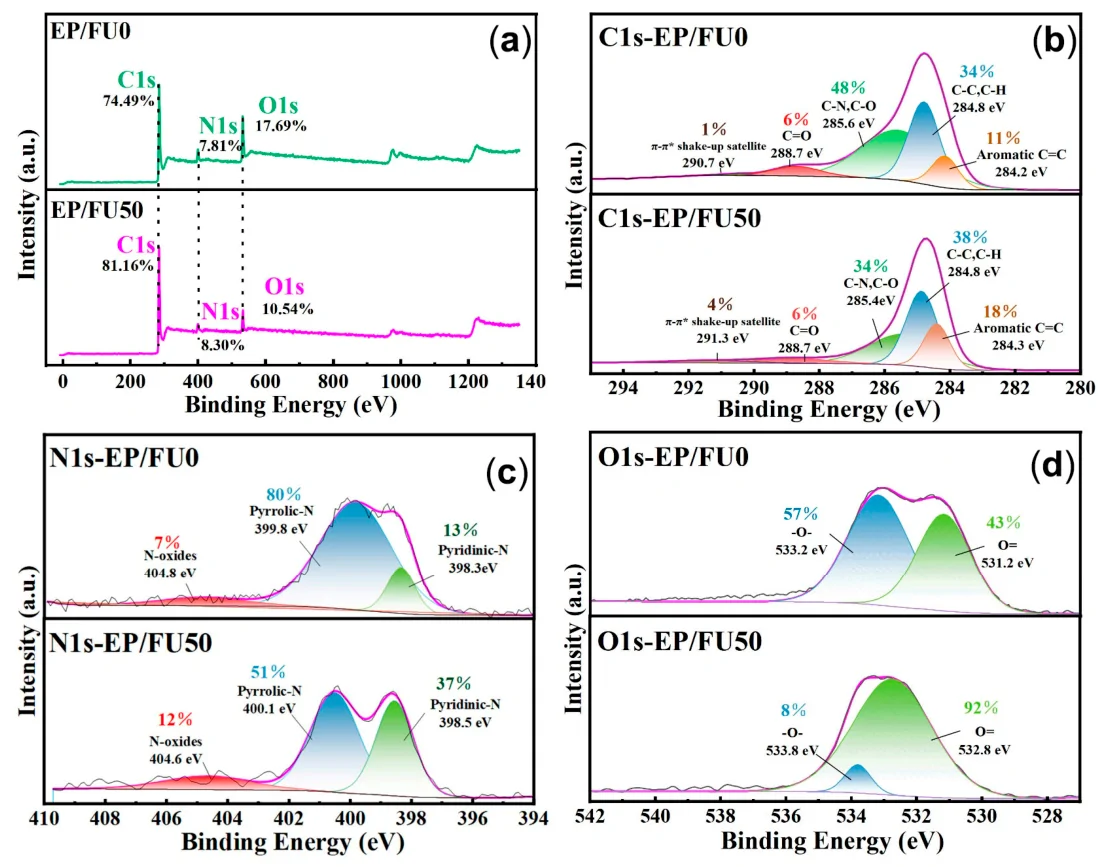
(**a**) XPS survey spectra and (**b**) C_1S_, (**c**) N_1S_, (**d**) O_1S_ spectra of the char residues of EP/FU0 and EP/FU40.

XPS spectra of the char residues after thermal degradation at 600 °C under air atmosphere are shown in Figure 11. The survey spectrum in Figure 11a reveals that the oxygen proportion in the char of EP/FU50 is markedly lower than that of EP/FU0. In contrast, the carbon proportion is higher. In the C1s spectra, the conjugated aromatic C=C peak at 284.2 eV and the π–π* shake-up satellite peak near 290 eV confirm abundant conjugated aromatic ring structures within the char. The peak at 288.7 eV is assigned to C=O groups, and the fractions of C=O species in the carbon framework are nearly identical for EP/FU0 and EP/FU50. The O1s spectra exhibit a counterintuitive result: the proportion of double-bonded oxygen (O=) in EP/FU50 char is significantly higher than that in EP/FU0. Combined with the above results, it can be inferred that the oxidized carbon species in EP/FU50 char exists mainly as C=O, while the oxidized species of EP/FU0 char contain a large amount of single-bonded C–O moieties.

In the N1s spectra, the peaks near 404.6 eV, 400 eV and 398.5 eV are attributed to pyridinic N-oxide, pyrrolic N (-N-) and pyridinic N (=N-), respectively. The pyridine ring possesses better thermal stability than the pyrrolic ring. More pyridinic structures were generated in EP/FU50 during thermal rearrangement, and the pyridinic N in residual char of EP/FU50 exhibits a lower oxidation ratio (the ratio of N-oxide to pyridinic-N is ~0.54 for EP/FU0 and ~0.32 for EP/FU50). It can be inferred that the dense char layer of EP/FU50 restricts the inward diffusion of oxygen, leading to less oxygen trapped within the char. The limited oxygen that penetrates into the char undergoes deep oxidation and mainly exists in the form of C=O groups.

Raman spectra of char residues for EP/FU0 and EP/FU50 are presented in Figure S5. The peak area ratio I_D_/I_G_ is widely adopted to characterize the graphitization degree of carbon materials. The I_D_/I_G_ value decreases with increasing FUPN loading, indicating the improved graphitization of char residues. Combined with the XPS results, it can be inferred that continuous, large-scale aromatic carbon skeletons are formed in the char layer of EP/FU50, which accounts for its high char yield under air atmosphere.

**Figure 12:**
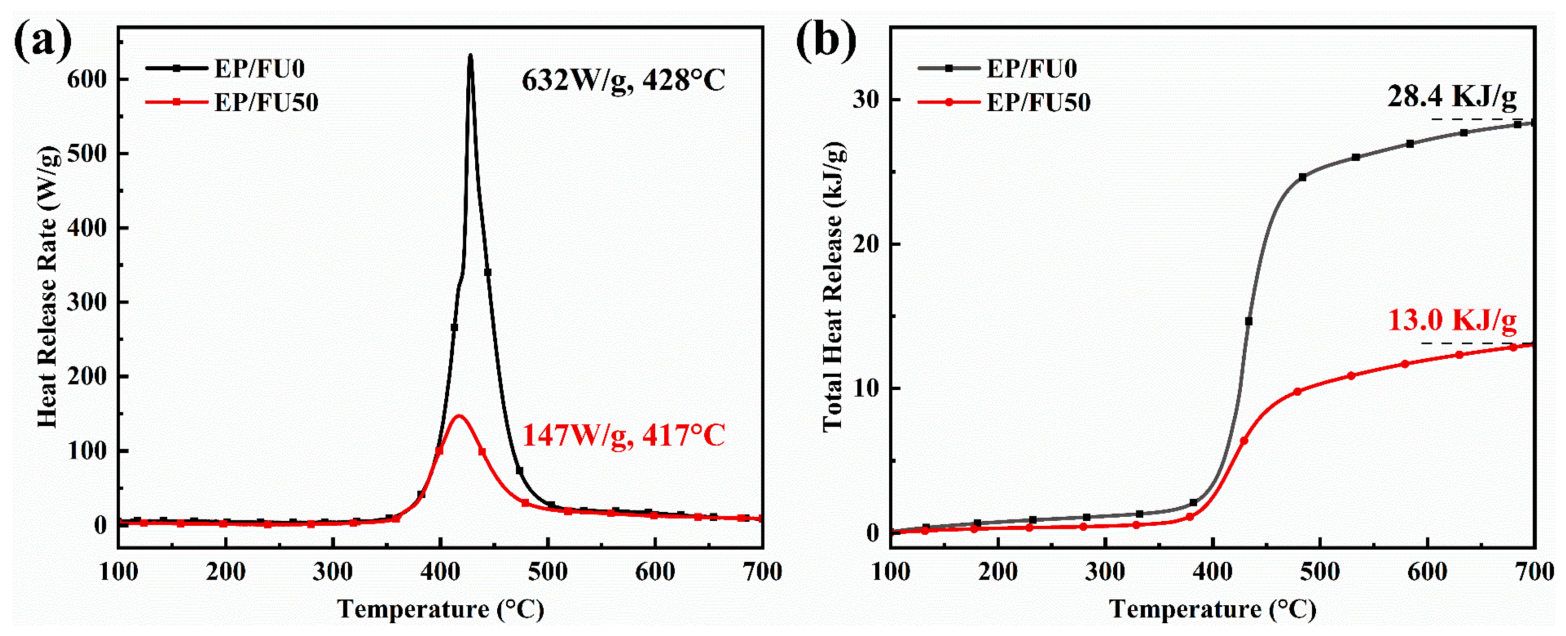
MCC results of EP/Fux: (**a**) heat release rate (HRR) as a function of temperature, (**b**) total heat release (THR) curves as a function of temperature.

Given the excellent thermal stability of EP/Fux systems, EP/FU50, which exhibited the optimal thermal stability, was selected as the representative sample to further investigate its flame-retardant performance via microscale combustion calorimetry (MCC). As shown in Figure 12, EP/FU0 displayed a sharp heat-release peak, with a peak heat release rate (pHRR) of 632 W/g at 428 °C. By contrast, the pHRR of EP/FU50 decreased markedly to 147 W/g at 417 °C, corresponding to a reduction of approximately 76.7%. The total heat release (THR) of EP/FU50 also declined from 28.4 kJ g^−1^ for EP/FU0 to 13.0 kJ g^−1^, with a reduction of around 54.2%. These results reveal that FUPN can effectively suppress the formation of combustible pyrolysis products and their heat release.

## 4. Conclusions

Currently, research on high-temperature-resistant bio-based polymers is rapidly advancing, while their modification effects on petroleum-based polymers have rarely been reported. In this work, bio-based phthalonitrile (FUPN) was employed as a reactive modifier for the widely used petroleum-based epoxy system DGEBF/DDS. The blend of FUPN and DGEBF/DDS exhibited low mixing viscosity, a broad processing window, and good reactivity upon heating. The FUPN/DGEBF/DDS system possessed good compatibility, and no obvious phase separation was observed in the cured resins. With increasing FUPN loading, both the *T*_g_ and thermal decomposition resistance of the cured materials were improved. Notably, the FUPN-modified epoxy system exhibited a more pronounced improvement in thermal stability under air (with a char yield of 31.5%). The improvement in the thermal stability could be attributed to the formation of continuous aromatic carbon skeletons with abundant quinone moieties. In summary, FUPN can enhance the thermal stability of cured resins while retaining satisfactory processability. This work demonstrates the great potential of FUPN as a bio-based modifier to improve the thermal performance of conventional petroleum-based epoxy resins.

## Figures and Tables

**Table 1:** Formulations of EP/FUx (DGEBF/DDS/FUPN) systems.

Sample	DGEBF (g)	DDS (g)	FUPN (g)
EP/FU0	10.000	3.725	0.000
EP/FU20	8.000	2.980	2.000
EP/FU30	7.000	2.607	3.000
EP/FU40	6.000	2.235	4.000
EP/FU50	5.000	1.862	5.000

**Table 2:** Dynamic mechanical analysis (DMA) data of the cured thermosets.

Sample	^a^ *E*′_25_ (MPa)	^b^ *E*′_100_ (MPa)	^c^ *R*_E_′ (%)	*T*_g_ (°C)	^d^ *E*′_r_ (MPa)	^e^$\;\nu_{e}$(mol/m^3^)
EP/FU0	2370	2056	86.75	161	10	864
EP/FU20	3119	2460	78.87	166	20	1709
EP/FU30	3488	2976	85.32	190	40	3252
EP/FU40	3576	3150	88.09	215	71	5494
EP/FU50	3542	3232	91.25	231	88	6605

Note: ^a^ Storage modulus at 25 °C; ^b^ storage modulus at 100 °C; ^c^ *R*_E_′ = *E*′_100_/*E*′_25_; ^d^ storage modulus measured at *T*_g_ + 30K; ^e^ apparent crosslink density.

**Table 3:** TGA results of EP/FUx under N_2_ atmosphere.

Sample	^a^ *T*_5%_ (°C)	^b^ *T*_max_ (°C)	^c^ *R*_max_ (%/min)	^d^ *C*_exp_ (%)	^e^ *C*_cal_ (%)	^f^ Δ*C* (%)
EP/FU0	362.0	395.9	14.3	21.4	/	/
FUPN	410.0	458.9	1.2	65.2	/	/
EP/FU20	361.0	393.5	7.8	41.0	30.2	10.8
EP/FU30	361.1	397.0	6.4	48.5	34.5	14.0
EP/FU40	369.7	400.7	4.5	55.7	38.9	16.8
EP/FU50	373.0	402.0	3.7	57.7	43.3	12.4

Note: ^a^ Temperature at 5% mass loss; ^b^ temperature at maximum mass loss; ^c^ maximum mass loss rate; ^d^ experimental char yield at 800 °C; ^e^ calculated char yield at 800 °C; ^f^ *C*_exp_–*C*_cal_.

**Table 4:** TGA parameters of cured EP/FUx resins under air atmosphere.

Sample	^a^ *T*_5%_ (°C)	^b^ *T*_1max_ (°C)	^c^ *R*_1max_ (%/min)	^d^ *T*_2max_ (°C)	^e^ *R*_2max_ (%/min)	^f^ *C*_800_ (%)
EP/FU0	312.6	328.5	4.0	546.9	6.2	3.4
EP/FU20	380.1	404.1	6.7	685.8	2.6	5.5
EP/FU30	379.3	397.1	6.6	738.3	2.4	13.1
EP/FU40	380.9	399.6	4.6	752.9	2.3	29.1
EP/FU50	387.8	399.5	2.8	751.8	2.0	31.5

Notes: ^a^ Temperature at 5% weight loss; ^b,c^ peak temperature and maximum mass-loss rate of the 1st degradation stage; ^d,e^ peak temperature and maximum mass-loss rate of the 2nd stage; ^f^ measured char yield at 800 °C.

**Table 5:** Comparison of thermal parameters obtained from air TGA for various heat-resistant epoxy resin.

Sample	*T*_5%_	^a^ *C*_800_	*T*_1max_	*T*_2max_	^a^ References
TGTS-PDA	368.0	15.4	423.5	669.1	[38]
E51/BPS-Ph/DDS	366.8	20.0	378.5	514.3	[39]
EP-TM5-DP5	383.5	20.0	373.8	547.0	[40]
EP-TP5-DP5	417.3	21.6	481.5	605.7	[40]
DGMPS/APDS	306	22	356	745	[41]
N2-O/DDS	391	22.2	437	/	[42]
^b^ DOPO-TA	299	32	351	<600	[43]
EP/FU50	387.8	31.5	399.5	751.8	This work

Note: ^a^ The data is derived from the optimal formulations in the relevant reference. ^b^ The *T*_g_ value of DOPO-TA in the reference is 119.2 °C.

## Data Availability

The original contributions presented in this study are included in the article/Supplementary Material. Further inquiries can be directed to the corresponding author.

## Supplementary Materials

The following supporting information can be downloaded at https://www.mdpi.com/article/10.3390/ma19204324/s1, Section S1: synthesis of FUPN. Section S2: Cure Kinetics. Figure S1. FTIR characterization of the raw materials and products; Figure S2. The DSC curves of the curing process of VAPN and FUPN; Figure S3. The DSC curves of various combination of DGEBF, DDS and FUPN (β = 10 K/min); Figure S4. The deconvoluted DSC curves for (a) EP/FU20, (b) EP/FU30, (c) EP/FU40, and (d) EP/FU50. at a heating rate of 10 K·min^−1^. Figure S5 Raman spectra of the char residues obtained from the cured materials after pyrolysis at 600 °C in air atmosphere; Table S1. Relative exothermic contributions of deconvoluted peaks 2 and 3 for EP/FUx blends.
