# Supplementary material for: Bio-Based Phthalonitrile Bearing a Schiff Base Moiety as a High-Performance Modifier for Epoxy Resins: Curing Behavior and Thermal Properties
Source: Materials (Basel). 2026 Oct 14;19(20):4324. doi: 10.3390/ma19204324 (PMC13707348; doi:10.3390/ma19204324)
Supplement: Supplementary file 1 [file materials-19-04324-s001.zip › materials-4607529-supplementary.pdf]

Bio-Based Phthalonitrile Bearing a Schiff Base Moiety as a High-Performance Modifier for Epoxy Resins: Curing Behavior and Thermal Properties

Cong Peng [1], Chenxu Liu [1], Zhanjun Wu [1,2,*]

[1] College of Fiber Engineering and Equipment Technology, Jiangnan University, Wuxi 214122, China; pc@jiangnan.edu.cn (C.P.); 6243017090@stu.jiangnan.edu.cn (C.L.)
[2] School of Materials Science and Engineering, Dalian University of Technology, Dalian 116024, China
* Correspondence: wuzhj@dlut.edu.cn

Supporting Information

Section S1: synthesis of FUPN

The intermediate VAPN was prepared as followed: Vanillin (15.22 g, 0.1 mol), anhydrous potassium carbonate (13.82 g, 0.1 mol) and 180 mL of DMF were added into a 500 mL three-necked flask. The mixture was stirred and heated to 70 °C under a nitrogen atmosphere for 0.5 h. Subsequently, 4-Nph (17.31 g, 0.1 mol) was charged, and the system was heated to 90 °C for another 6.5 h. Upon completion of the reaction, the reaction solution was slowly poured into 1500 mL of deionized water under stirring, resulting in substantial precipitate formation. The mixture was kept in an ice-water bath for 5 h before filtration. The filter cake was re-dispersed in deionized water, allowed to stand, and filtered under reduced pressure; this washing procedure was repeated 3–5 times with deionized water. The product was dried in a vacuum oven at 55 °C for 24 h to afford VAPN (25.87 g, yield: 93.2%).

^{1}H NMR(400 MHz,DMSO-d6) δ 10.02 (s, 1H), 8.08 (d, J = 8.8 Hz, 1H), 7.78 (d, J = 2.6 Hz, 1H), 7.71 (d, J = 1.8 Hz, 1H), 7.65 (dd, J = 8.1, 1.8 Hz, 1H), 7.44 (d, J = 8.1 Hz, 1H), 7.36 (dd, J = 8.8, 2.6 Hz, 1H), 3.83 (s, 3H). **^{13}C NMR** (101 MHz, DMSO) δ 192.02, 160.35, 151.49, 146.23, 136.19, 135.00, 124.36, 122.63, 121.75, 121.18, 116.62, 115.85, 115.33, 112.92, 108.38, 56.06.

VAPN (27.83 g, 0.1 mol), anhydrous sodium acetate (8.20 g, 0.1 mol) and 250 mL of anhydrous ethanol were added to a three-necked flask under a nitrogen atmosphere. The mixture was heated to 70 °C and stirred for 0.5 h. Subsequently, 8.85 mL of furfurylamine was added in one portion, and the reaction temperature was increased to 80 °C for 2.5 h. The reaction progress was monitored by thin-layer chromatography (TLC). Upon completion of the reaction, the three-necked flask was transferred to a refrigerator maintained at 5 °C and kept undisturbed for 12 h. The solid precipitate was collected by vacuum filtration and washed with cold ethanol 2–3 times. The product was dried in a vacuum oven at 50 °C for 24 h to afford FUPN (33.61 g, yield: 94.0%).

^{1}H NMR (400 MHz, DMSO-d6) δ 8.48 (s, 1H), 8.04 (d, J = 8.8 Hz, 1H), 7.71 (d, J = 2.6 Hz, 1H), 7.63 – 7.53 (m, 2H), 7.42 (dd, J = 8.2, 1.8 Hz, 1H), 7.37 – 7.21 (m, 2H), 6.43 (dd, J = 3.2, 1.8 Hz, 1H), 6.36 (d, J = 3.2 Hz, 1H),

4.77 (s, 2H), 3.77 (s, 3H). **^{13}C NMR** (101 MHz, DMSO) δ 161.93, 160.92, 152.30, 151.22, 143.15, 142.50, 136.11, 135.00, 122.51, 122.28, 121.27, 120.65, 116.53, 115.91, 115.37, 111.62, 110.53, 107.85, 107.51, 56.21, 55.81.

Section S2: Cure Kinetics

In this work, the Starink isoconversional method was employed to calculate parameters such as activation energy during the crosslinking of FUPN. The conversion α was determined by integrating the heat flow from DSC measurements, as defined in Equation S1. Herein, ΔH_T is the cumulative heat release upon heating to temperature T, and ΔH0 represents the total heat release over the entire reaction range.

$$\alpha = \frac{\Delta H_T}{\Delta H_0}$$

Eq.S1

The reaction rate expression was further established based on conversion. Following the general form of thermal analysis kinetics, the curing rate can be expressed as the product of a temperature-dependent term $k(T)$ and a conversion function $f(\alpha)$, as shown in Equation S2. This expression serves as the basis for separately determining the apparent activation energy and reaction model in the subsequent analysis.

$$\frac{d\alpha}{dt} = k(T)f(\alpha)$$

Eq.S2

Assuming that $k(T)$ follows the Arrhenius relationship, it can be expressed as Equation S3. Herein, A is the pre-exponential factor, E denotes the apparent activation energy, R is the gas constant, and T represents the absolute temperature.

$$k(T) = A\exp\left(-\frac{E}{RT}\right)$$

Eq.S3

The experiments were carried out under constant heating-rate rather than isothermal conditions. Therefore, the heating rate β=dT/dt was introduced to transform the time variable into a temperature variable. Substituting Equation S3 into Equation S2 and performing this variable transformation yields the non-isothermal rate equation shown in Equation S4. With this treatment, DSC data collected at multiple heating rates can be used for isoconversional energy-barrier analysis.

$$\frac{d\alpha}{dT} = \frac{A}{\beta}\exp\left(-\frac{E}{RT}\right)f(\alpha)$$

Eq.S4

After obtaining the α-T data at different heating rates, the Starink isoconversional relationship was used to calculate the apparent activation energy E_α at a given conversion, as shown in Equation (5). Herein, T_α denotes the absolute temperature required to reach conversion α. For each fixed α, the plot of $\ln(\beta/T_\alpha^{1.92})$ versus $1/T_\alpha$ was linear fitted, from which the evolution of E_α with conversion can be obtained.

$$\ln\left(\frac{\beta}{T_\alpha^{1.92}}\right) = \text{constant} - \frac{1.0008E_\alpha}{RT_\alpha}$$

Eq.S5

Section S3.Figueres

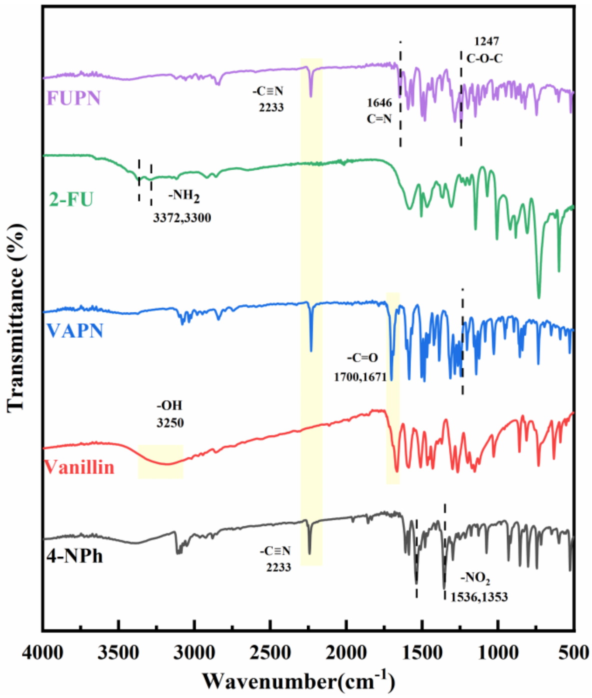

Figure S1.FTIR characterization of the raw materials and products

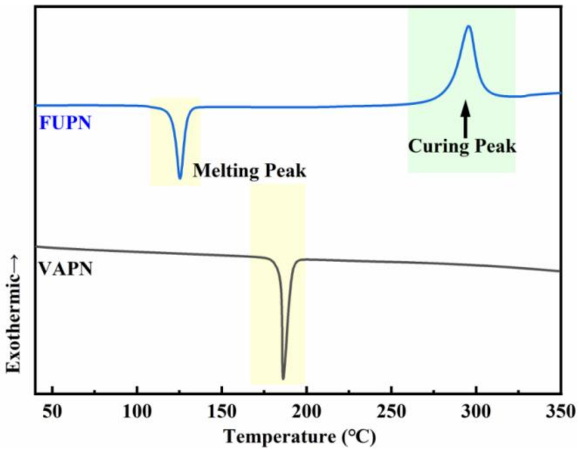

Figure S2. The DSC curves of the curing process of VAPN and FUPN

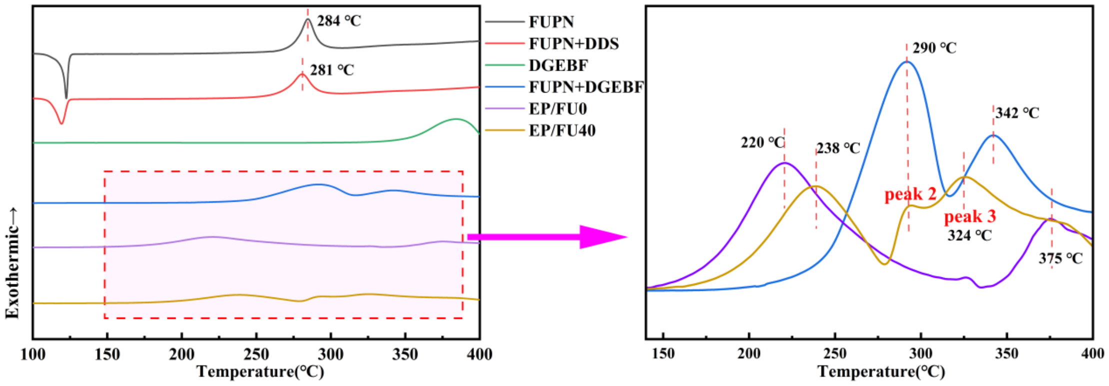

Figure S3. The DSC curves of various combination of DGEBF, DDS and FUPN（β=10K/min）

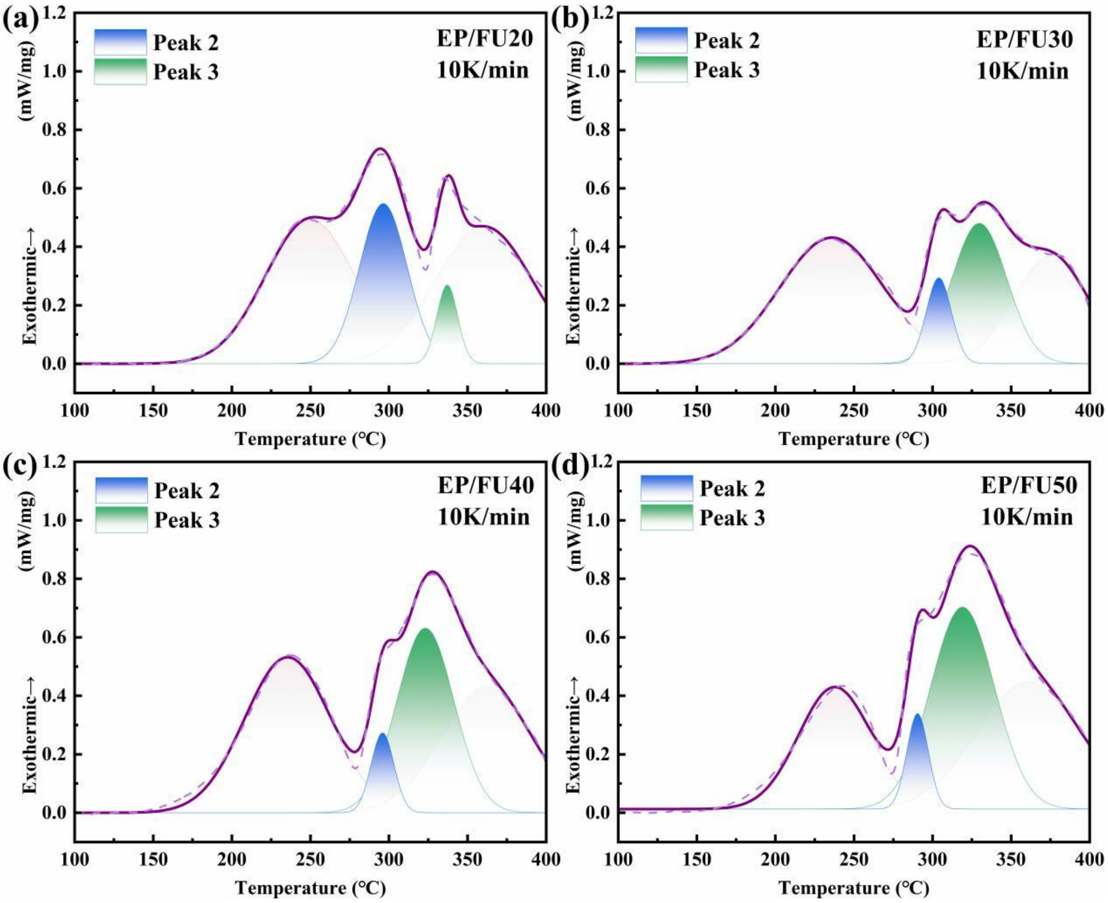

Figure S4. The deconvoluted DSC curves for (a) EP/FU20, (b) EP/FU30, (c) EP/FU40, and (d) EP/FU50 at a heating rate of 10 K·min^{-1}:

Table S1. Relative exothermic contributions of deconvoluted peaks 2 and 3 for EP/FUx blends.

Sample	Peak 2 area (mW K mg^{-1})	Peak 3 area (mW K mg^{-1})	Peak 2 contribution(%)	Peak 3 contribution(%)
EP/FU20	20.8	4.5	82.2	17.8
EP/FU30	5.9	21.1	21.9	78.1
EP/FU40	5.0	28.8	14.8	85.2
EP/FU50	5.6	35.1	13.7	86.2

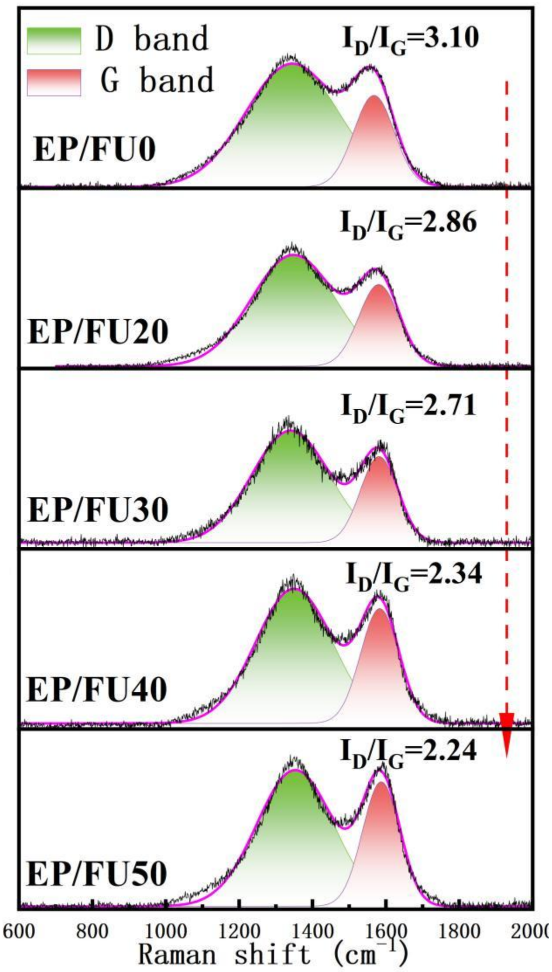

Figure S5 Raman spectra of the char residues obtained from the cured materials after pyrolysis at 600 °C in air atmosphere.
